# A Biotransformation Process for Production of Genistein from Sophoricoside by a Strain of *Rhizopus oryza*

**DOI:** 10.1038/s41598-019-42996-z

**Published:** 2019-04-25

**Authors:** Jianfeng Mei, Xiang Chen, Jianghua Liu, Yu Yi, Yanlu Zhang, Guoqing Ying

**Affiliations:** 1 College of Pharmaceutical Science, Zhejiang University of Technology, Hangzhou, 310014 China; 2 Zhoushan Institute for Food and Drug Control, Zhoushan, 316021 China

**Keywords:** Industrial microbiology, Biocatalysis

## Abstract

Genistein is known to have multiple biological activities and has great potential for use as a preventative medicine and in disease treatment. Genistein can be extracted from plants, but also can be obtained from its glycoside form, sophoricoside, which is more abundant in some plants. Biotransformation by unpurified microbial enzymes has the advantage of low cost and is a preferred method for production of natural compounds. This study isolated a strain of *Rhizopus oryzae* that could produce *β*-glucosidase, which efficiently hydrolyzes sophoricoside into genistein, from an enrichment culture of the dried fruits of *Sophora japonica*. After the composition of enzyme-producing medium and biotransformation conditions were optimized, a genistein yield of 85.6% was obtained after 24 h in a shake-flask biotransformation at pH 7.0 using an initial substrate concentration of 1 g/L. The developed process provides an alternative method for production of genistein, and would be suitable for scale-up production in the pharmaceutical industry.

## Introduction

Genistein [4,5,7-trihydroxyisoflavone or 5,7-dihydroxy-3-(4-hydroxyphenyl) chromen-4-one] was originally identified and isolated from dyer’s broom (*Genista tinctorial*)^1^. Genistein has a chemical structure similar to estradiol, and is well known as a phytoestrogen^2^. It has been researched in regard to its effect on the reduction of menopausal symptoms and reduction of cardiovascular risk factors in osteopenic, post-menopausal women^3^. In recent years, some studies have found that genistein has additional physiological functions, such as antioxidant, anti-inflammatory, anti-osteoclastic^4^, anticancer^5^, and antiobesity^6^ activities. Genistein has great potential for use as a preventative medicine and in disease treatment.

The glycoside form of genistein (genistein 4′-β-d-glucoside), namely sophoricoside, has similar physiological function to genistein, but its potency in some activities is far lower than that of genistein^7^. Studies have shown that sophoricoside exhibits pharmacological activities only after it is metabolized into genistein *in vivo*^8,9^. The content of sophoricoside is higher than genistein in a number of plants, but especially in *Sophora japonica* in which their content differ more than tenfold^10^. Sophoricoside has a wider source base and is cheaper than genistein. Therefore, it is logical that large quantities of genistein should be obtained from sophoricoside by hydrolysis. Production of genistein from sophoricoside has been reported, including biotransformation with β-glucosidase^11^, microbial cells^12,13^, along with acid hydrolysis of sophoricoside^14^. Given that biotransformation can provide excellent specificity and is environmentally friendly, it is a preferred method for the preparation of many natural compounds. However, when purified enzyme is used in biotransformation, this method incurs the disadvantage of high cost. Direct use of unpurified enzymes for biotransformation can avoid this shortcoming.

**Figure 1:**
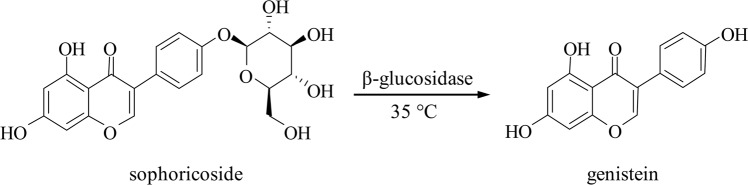
Biotransformation of sophoricoside into genistein by β-glucosidase.

In this study, a strain of *Rhizopus oryzae* was isolated from an enrichment culture of Sophorae Fructus to produce β-glucosidase, which was used for biotransformation of sophoricoside into genistein. The biotransformation scheme is illustrated in Fig. 1. The composition of the enzyme-producing medium was optimized for *Rhizopus oryzae* to produce β-glucosidase, and the enzymatic hydrolysis conditions were optimized to improve the yield of genistein. This biotransformation process has great potential for use in the pharmaceutical industry.

## Results and Discussion

### Isolation of microbial strains for biotransformation

**Figure 2:**
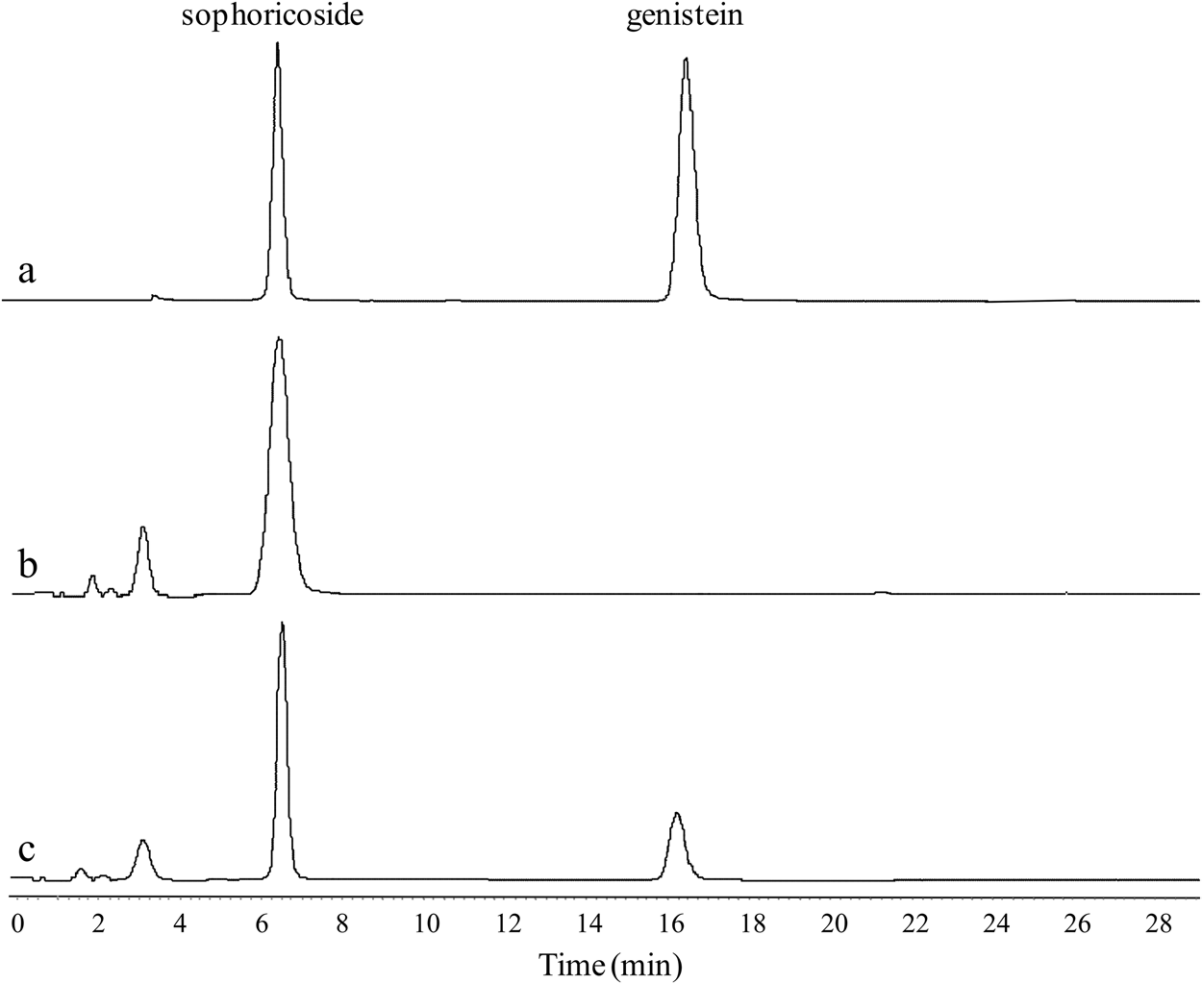
HPLC-UV analysis (at 260 nm) of sophoricoside and genistein. (**a**) Standard sophoricoside and genistein, (**b**) Incubation of sophoricoside in blank medium, (**c**) Biotransformation of sophoricoside by β-glucosidase from *Rhizopus oryzae* LJH-3.

Among ten fungi strains that were isolated from the enrichment culture of Sophorae Fructus, only one strain, LJH-3, produced enzymes that converted sophoricoside into genistein with a yield over 20%. Figure 2c shows that about 25% of sophoricoside was converted into genistein. Given the equimolar relationship between the product and substrate, it was apparent that genistein was the only product. Therefore, we concluded that strain LJH-3 secretes β-glycosidase, which trimmed the glycosidic bond of sophoricoside to produce genistein. The addition of sophoricoside into the culture from some other fungal strains, the sophoricoside concentration decreased obviously, but genistein was could not be detected by HPLC (data not shown). It suggests that the nucleus structure of isoflavone in sophoricoside and genistein was destroyed by some hydrolytic enzymes from these strains.

**Figure 3:**
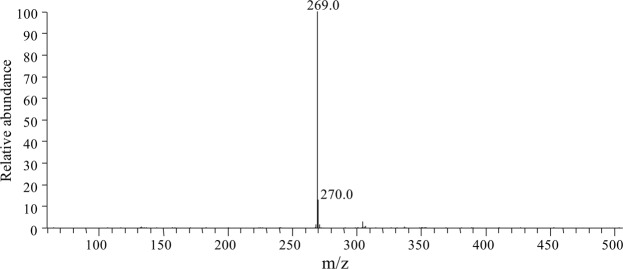
LC–ESI-MS spectra of the biotransformation product from sophoricoside.

HPLC analysis of the LJH-3 biotransformation mixture (Fig. 2c) revealed a peak with the same retention time as genistein. Mass spectral analysis of the peak by ESI–MS showed the major ion to be [M–H]^−^ at *m*/*z* 269 (Fig. 3), indicating that the product has a molecular weight of 270 Da. This information again illustrates that the biotransformation product is genistein.

Based on morphological features and ITS1-5.8S-ITS2 sequence analysis, strain LJH-3 was identified as a strain of *Rhizopus oryzae*. It was deposited at the Guangdong Microbial Culture Center, China, under the identifier GDMCC No. 601450.

The β-glucosidase activity produced by LJH-3 under nonoptimized enzyme-producing medium was 0.216 U/mL.

### Optimization of enzyme-producing medium

Taking β-glucosidase activity as an index, the composition of the enzyme-producing medium for LJH-3 was optimized by single-factor experiments and response surface methodology (data available at supplementary information). After the compostion of enzyme-producing medium by strain LJH-3 were optimized, The activity of β-glucosidase was significantly increased. The conditions suitable for the growth of LJH-3 and the production of glucosidase were: maltose, 10.8 g/L; yeast extracts, 12 g/L; peptone, 7.1 g/L; NaCl, 5 g/L; MgSO_4_, 1.0 g/L; initial pH, 7.0; temperature, 30 °C; speed of rotary shaker, 200 rpm; duration, 6 days. Under these cultivation conditions, the β-glucosidase activity from strain LJH-3 was 1.16 U/mL, which is 5.37 times higher than the activity observed before optimization (0.216 U/mL). Using this enzyme broth to convert sophoricoside at a concentration of 10 mg/L, the yield of genistein was 91.1%.

### Optimization of biotransformation process

#### Substrate concentration

**Figure 4:**
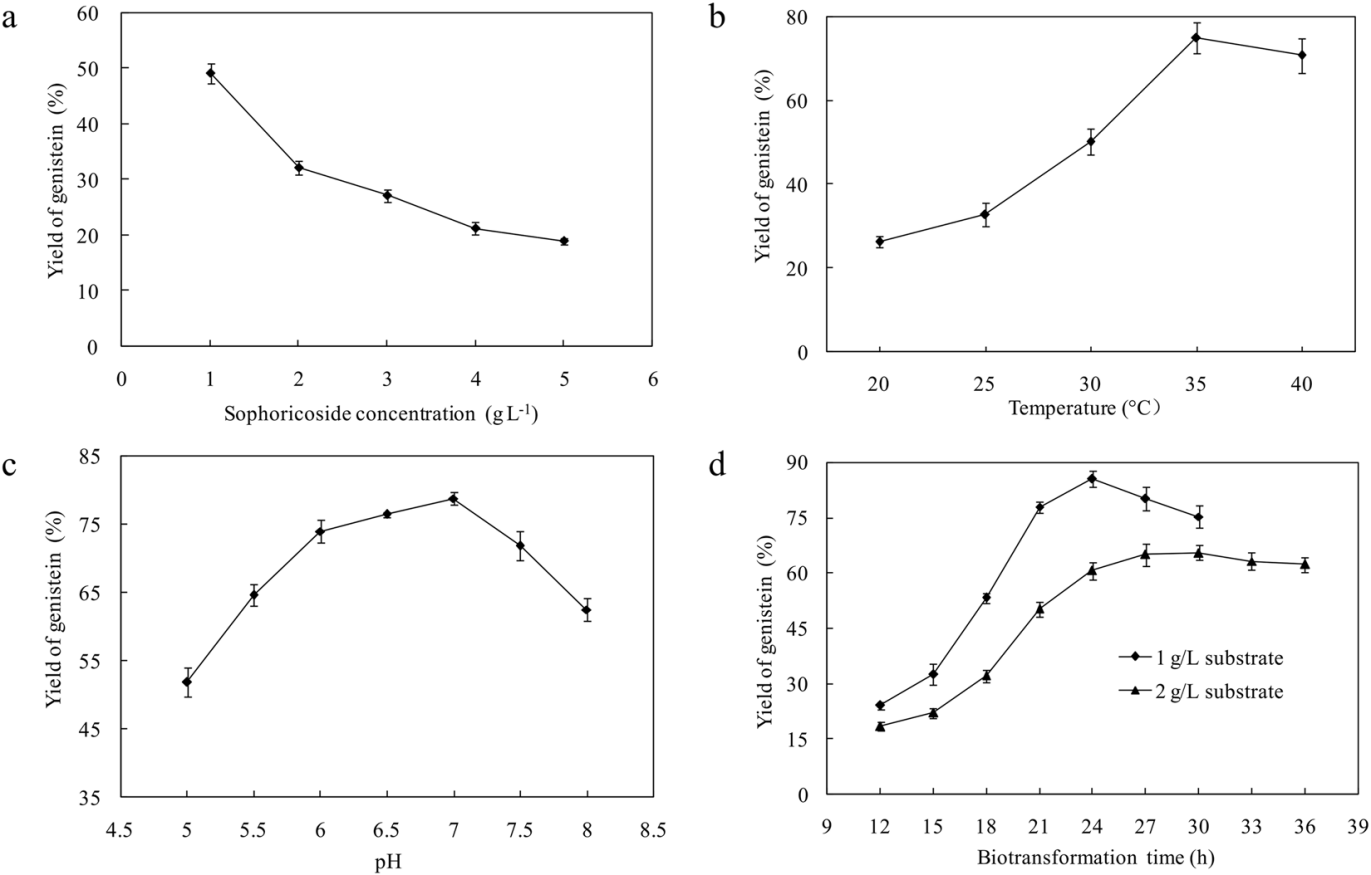
Optimization of biotransformation conditions to improve the yield of genistein from sophoricoside by β-glucosidase from *Rhizopus oryzae* LJH-3: (**a**) Sophoricoside concentration, (**b**) Temperature, (**c**) pH, and (**d**) Biotransformation time. Results are expressed as mean ± standard deviation (*n* = 3).

The effects of the main factors in the biotransformation process (substrate concentration, temperature, pH and biotransformation time) on the genistein yield were investigated. The biotransformation was carried out at 30 °C for 20 h when the sophoricoside concentration was set at 1–5 g/L. With the increase of the substrate concentration, the yields of genistein decreased significantly (Fig. 4a). Though the yield of genistein was only 49.3% at the substrate concentration of 1 g/L, prolonging the biotransformation time can improve the yield. So the concentration of sophoricoside was set at 1 g/L for further optimization.

#### Temperature

The biotransformation was incubated at different temperatures ranging from 20 to 40 °C to examine the effect of temperature on genistein yield. The maximum yield of genistein (84.1%) was obtained at 35 °C. As shown in Fig. 4b, the effect of temperature on the yield of genistein is highly significant. Although the temperature increased by only 5 °C from 30 °C, the yield of genistein increased by nearly 25 percentage points.

#### pH of biotransformation system

After cultivation of the *Rhizopus oryzae* enzyme broth, the pH of the broth was slightly acidic (pH ~6.0) and unsuitable for dissolution of sophoricoside, which is easily dissolved in weakly alkaline water. To test the effect of broth pH on the biotransformation, the pH was adjusted to 5.0–8.0 using NaOH or HCl solution. The yields of genistein at various pH values are shown in Fig. 4c. The maximum yield of 84.2% was observed at pH 7.0. This pH indicates a compromise between optimal enzyme activity and sophoricoside solubility.

#### Biotransformation time

The biotransformations were performed using the sophoricoside concentration of 1 g/L and 2 g/L at pH 7.0 on a rotary shaker at 200 rpm and 28 °C. A fraction of the biotransformation sample was removed every 3 h for analysis. When the sophoricoside concentration was 1 g/L, the maximum yield of genistein (85.6%) was achieved after 24 h (Fig. 4d). If the biotransformation time extended past 24 h, the yield of genistein dropped slightly, which may indicate hydrolysis of genistein by hydrolytic enzymes in the broth. When the sophoricoside concentration was 2 g/L, the maximum yield of genistein (65.5%) was achieved after 30 h. prolonging the biotransformation time can improve the yield of genistein.

## Conclusions

A biotransformation process was developed in which a fungal enzyme extract was used to convert sophoricoside into genistein. From the enrichment culture of the dried fruits of *Sophora japonica*, a fungal strain with the ability to produce β-glucosidase was isolated and identified as a *Rhizopus oryzae*. After the composition of enzyme-producing medium and biotransformation conditions was optimized, a maximum genistein yield reached 85.6% using a sophoricoside concentration of 1 g/L. Compared with the biotransformation using purified β-glucosidase or acid hydrolysis of sophoricoside to produce genistein, the advantages of the current technique are obvious, such as the lower cost of enzyme, no by-products, and high yield. This method has the potential for use as an alternative means of genistein production in the pharmaceutical industry and would be suitable for scale-up production.

## Methods

### Materials and chemicals

Sophoricoside, genistein, *p*-nitrophenol (pNP), and *p*-nitrophenyl-β-d-glucopyranoside (pNPG) with 98% purity was purchased from Shanghai Aladdin Biochemical Technology (Shanghai, China). Sophorae Fructus (Chinese medicine name: *Huaijiao*), dried fruits of *Sophora japonica* L, were purchased from XuFang Chinese Herbal Medicine Management (Anguo, Hebei, China). HPLC grade methanol were supplied by Merck (Shanghai, China). All other reagents used in the study were of analytical or biochemical grade.

### Isolation and selection of microorganism

50 g of dried powder of Sophorae Fructus was loaded in a 250-mL Erlenmeyer flask and soaked with sterile water. After 2 days incubation at 30 °C, the enrichment culture was suspended and diluted by sterile water, the dilution was plated onto potato dextrose agar (PDA). After 2 days incubation at 30 °C, single fungal colonies were picked and transferred onto a fresh PDA plate repeatedly until a pure culture was obtained. The isolated strains were maintained on PDA slants for further studies.

The morphological identification of the strain was observed under an Olympus CX32 light microscope (Olympus, Tokyo, Japan). Extraction of genomic DNA, polymerase chain reaction (PCR) amplification of ITS1-5.8S-ITS2 sequence, and sequencing of the purified PCR products were completed by Sangon Biotech Co., Ltd. (Shanghai, China). The sequences were compiled and compared with the sequences in the NCBI database using the BLAST (Basic Local Alignment Search Tool) program. Construction of a neighbor joining tree was conducted using the MEGA 7.0 software^15^.

### β-Glucosidase activity assay

The β-glucosidase activity was assayed using the procedure reported by Watanabe^16^ with slight modifications. Several concentrations of pNP solution were used to construct a standard curve. The β-glucosidase activity was measured at 30 °C with 5 mM pNPG as the substrate in 0.4 mL of 20 mM phosphate buffer (pH 6.0) and with 0.4 mL of enzyme preparation. After incubation for 60 min, the reaction was stopped by adding 2.5 mL of 1 M Na_2_CO_3_, and the release of pNP was measured at A_400_. The results were calculated using the equation obtained from the standard curve. One unit of β-glucosidase activity was defined as the amount of enzyme that yielded 1 μmol of pNP per minute under the settled experimental conditions.

### Biotransformation of sophoricoside

100 mL of enzyme-producing medium in a 250-mL flask was inoculated with fungal mycelia. The culture was cultivated for 3 days in a rotary shaker at 30 °C and 200 rpm, and then was pooled out and filtered. The filtrate was transferred to another flask, and sophoricoside dissolved in 1 ml of weakly alkaline water was added into the culture. The mixture was incubated for 20 h under the same conditions.

Enzyme-producing medium consisted of sucrose (30 g/L), NH_4_Cl (4 g/L), NaCl (5 g/L), K_2_HPO_4_ (1 g/L), and MgSO_4_ (0.5 g/L) at natural pH.

### Analytical methods

Biotransformation broth was twice extracted with an equivalent volume of ethyl acetate, and all the organic layers were combined. After that, the extracted solutions were concentrated under vacuum condition at 40 °C. Finally, the collected residues were dissolved in methanol and filtered through 0.22 μm millipore filter, then analyzed by RP-HPLC. The HPLC analysis was conducted on a Shimadzu SPD-20A chromatograph (Shimadzu, Kyoto, Japan) equipped with a Phenomenex Luna C18 column (5 µm, 4.6 × 250 mm). The mobile phase was composed of methanol–water (contains 0.15% formic acid) in the ratio 60:40 (v:v). The wavelength was set at 260 nm.

Genistein produced by biotransformation was confirmed by liquid chromatography–electrospray ionization mass spectrometry (LC–ESI-MS) using an Agilent 6210 TOF LC/MS (Agilent, Santa Clara, CA, USA).

### Data collection and analysis

All the treatments used for analyses were performed in triplicates., and results are reported as mean ± standard deviation. The yield of genistein was calculated as follows:

$${\rm{Genistein}}\,\mathrm{yield}( \% )=\frac{{\rm{Weight}}\,{\rm{of}}\,{\rm{genistein}}/{\rm{MW}}\,{\rm{of}}\,{\rm{genistein}}}{{\rm{Weight}}\,{\rm{of}}\,{\rm{sophoricoside}}/{\rm{MW}}\,{\rm{of}}\,{\rm{sophoricoside}}}$$

where MW is the molecular weight (MW of genistein is 270.2; MW of sophoricoside is 432.4).

## Supplementary information

Data on Optimization of Enzyme-producing Medium
