# Supplementary material for: A Biotransformation Process for Production of Genistein from Sophoricoside by a Strain of Rhizopus oryza
Source: Sci Rep. 2019 Apr 25;9:6564. doi: 10.1038/s41598-019-42996-z (PMC6484079; doi:10.1038/s41598-019-42996-z)
Supplement: Supplementary file 1 — Data on Optimization of Enzyme-producing Medium [file 41598_2019_42996_MOESM1_ESM.docx]

**A Biotransformation Process for Production of Genistein from Sophoricoside by a Strain of *Rhizopus oryza***

Jianfeng Mei^1^, Xiang Chen^2^, Jianghua Liu^1^, Yu Yi^1^, Yanlu Zhang^1^ & Guoqing Ying^1^

^1^ College of Pharmaceutical Science, Zhejiang University of Technology, Hangzhou 310014, China

^2^ Zhoushan Institute for Food and Drug Control, Zhoushan 316021, China

Corresponding author: Guoqing Ying

E-mail: bioph@zjut.edu.cn

Phone & Fax: +86-571-88871029

Address: 18 Chaowang Road, Xiacheng District, Hangzhou 310014, China

Data on Optimization of Enzyme-producing Medium

**1. Carbon source**

| Carbon source  (10 g/L) | β-Glucosidase activity (U/mL) | | | | |
| --- | --- | --- | --- | --- | --- |
|  | 1 | 2 | 3 | Average | STDEV |
| Glucose | 0.277 | 0.294 | 0.286 | 0.285 | 0.008748 |
| **Maltose** | **0.332** | **0.364** | **0.331** | **0.342** | **0.018675** |
| Sucrose | 0.297 | 0.284 | 0.306 | 0.295 | 0.011154 |
| Lactose | 0.172 | 0.173 | 0.164 | 0.170 | 0.004579 |
| Starch | 0.166 | 0.176 | 0.170 | 0.171 | 0.004867 |

.

The optimal carbon source is maltose.

**2. Maltose concentration**

| Maltose concentration (g/L) | β-Glucosidase activity (U/mL) | | | | |
| --- | --- | --- | --- | --- | --- |
|  | 1 | 2 | 3 | Average | STDEV |
| 5 | 0.193 | 0.220 | 0.193 | 0.202 | 0.015399 |
| **10** | **0.352** | **0.367** | **0.331** | **0.350** | **0.018143** |
| 20 | 0.345 | 0.334 | 0.361 | 0.347 | 0.013334 |
| 30 | 0.337 | 0.309 | 0.320 | 0.322 | 0.014242 |
| 40 | 0.292 | 0.317 | 0.287 | 0.299 | 0.016317 |

The optimal maltose concentration is 10 g/L.

**3. Nitrogen source**

| Code | Nitrogen source | β-Glucosidase activity (U/mL) | | | | |
| --- | --- | --- | --- | --- | --- | --- |
|  |  | 1 | 2 | 3 | Average | STDEV |
| A | Beef extracts (10 g/L) | 0.329 | 0.293 | 0.282 | 0.301 | 0.024685 |
| B | Peptone (10 g/L) | 0.315 | 0.325 | 0.294 | 0.311 | 0.015451 |
| C | Yeast extracts (10 g/L) | 0.751 | 0.721 | 0.659 | 0.710 | 0.047244 |
| D | Beef extracts (5 g/L)+peptone (5 g/L) | 0.704 | 0.653 | 0.640 | 0.666 | 0.03359 |
| E | Beef extracts (5 g/L)+yeast extracts (5 g/L) | 0.737 | 0.693 | 0.708 | 0.713 | 0.02251 |
| **F** | **Peptone (5 g/L)+yeast extracts(5 g/L)** | **0.915** | **0.842** | **0.837** | **0.865** | **0.043465** |
| G | Ammonium sulphate (10 g/L) | 0.184 | 0.171 | 0.168 | 0.175 | 0.008371 |
| H | Sodium nitrite (10 g/L) | 0.115 | 0.151 | 0.136 | 0.134 | 0.018178 |

The optimal nitrogen source is peptone (5 g/L)+yeast extracts(5 g/L).

**4. Peptone concentraton**

| Peptone concentraton (g/L) | β-Glucosidase activity (U/mL) | | | | |
| --- | --- | --- | --- | --- | --- |
|  | 1 | 2 | 3 | Average | STDEV |
| 3 | 0.707 | 0.675 | 0.712 | 0.698 | 0.019653 |
| **6** | **0.786** | **0.825** | **0.818** | **0.810** | **0.020606** |
| 9 | 0.785 | 0.803 | 0.774 | 0.787 | 0.014444 |
| 12 | 0.783 | 0.805 | 0.775 | 0.787 | 0.015444 |
| 15 | 0.781 | 0.740 | 0.790 | 0.770 | 0.026891 |

The optimal peptone concentraton is 6 g/L.

**5. Yeast extracts concentraton**

| Yeast extracts concentraton (g/L) | β-Glucosidase activity (U/mL) | | | | |
| --- | --- | --- | --- | --- | --- |
|  | 1 | 2 | 3 | Average | STDEV |
| 3 | 0.723 | 0.717 | 0.691 | 0.710 | 0.017192 |
| 6 | 0.820 | 0.827 | 0.809 | 0.819 | 0.009109 |
| **9** | **0.871** | **0.902** | **0.897** | **0.890** | **0.016527** |
| 12 | 0.872 | 0.907 | 0.902 | 0.894 | 0.018523 |
| 15 | 0.885 | 0.906 | 0.888 | 0.893 | 0.011699 |

The optimal Yeast extracts concentraton is 9 g/L.

**6. Inorganic salt**

| Inorganic salt  (1 g/L) | β-Glucosidase activity (U/mL) | | | | |
| --- | --- | --- | --- | --- | --- |
|  | 1 | 2 | 3 | Average | STDEV |
| MnSO_4_ | 0.833 | 0.836 | 0.858 | 0.842 | 0.01403852 |
| **MgSO_4_** | **0.941** | **0.911** | **0.924** | **0.925** | **0.01460369** |
| ZnSO_4_ | 0.879 | 0.858 | 0.857 | 0.865 | 0.01246233 |
| KH_2_PO_4_ | 0.848 | 0.849 | 0.878 | 0.858 | 0.01683265 |
| CuSO_4_ | 0.781 | 0.760 | 0.752 | 0.765 | 0.01511786 |
| Control | 0.854 | 0.877 | 0.875 | 0.868 | 0.01283816 |

The addition of 1 g/L MgSO_4_ can improve β-glucosidase activity.

**7. MgSO_4_ concentration**

| MgSO_4_ concentration (g/L) | β-Glucosidase activity (U/mL) | | | | |
| --- | --- | --- | --- | --- | --- |
|  | 1 | 2 | 3 | Average | STDEV |
| **1** | **0.906** | **0.936** | **0.935** | **0.926** | **0.017013** |
| 2 | 0.940 | 0.918 | 0.938 | 0.932 | 0.012168 |
| 3 | 0.929 | 0.896 | 0.935 | 0.920 | 0.021271 |
| 4 | 0.885 | 0.922 | 0.904 | 0.904 | 0.018648 |
| 5 | 0.821 | 0.884 | 0.892 | 0.865 | 0.039044 |

The optimal MgSO_4_ concentration is not higher than 9 g/L.

8. pH

| pH | β-Glucosidase activity (U/mL) | | | | |
| --- | --- | --- | --- | --- | --- |
|  | 1 | 2 | 3 | Average | STDEV |
| 5.0 | 0.872 | 0.914 | 0.907 | 0.898 | 0.022361 |
| 6.0 | 1.115 | 1.107 | 1.086 | 1.103 | 0.014952 |
| **7.0** | **1.112** | **1.109** | **1.143** | **1.121** | **0.018587** |
| 8.0 | 1.131 | 1.100 | 1.111 | 1.114 | 0.015648 |
| 9.0 | 1.042 | 1.061 | 1.026 | 1.043 | 0.017512 |

The optimal pH is 7.0.

**9. Optimization by Response Surface Methodology**

**Tab. 1** Coded levels and the variables used in the Box-Behnken Design

| Factors | Code | Actual levels of coded factors | | |
| --- | --- | --- | --- | --- |
|  |  | -1 | 0 | 1 |
| Yeast extracts (g/L) | X_1_ | 6 | 9 | 12 |
| Peptone (g/L) | X_2_ | 3 | 6 | 9 |
| Maltose (g/L) | X_3_ | 5 | 10 | 15 |

Tab. 2 The Box–Behnken design of the variables and experimental results

| 试验号 | X1 | X2 | X3 | Y |
| --- | --- | --- | --- | --- |
| 1 | -1 | -1 | 0 | 0.522 |
| 2 | 1 | -1 | 0 | 0.862 |
| 3 | -1 | 1 | 0 | 0.653 |
| 4 | 1 | 1 | 0 | 0.984 |
| 5 | -1 | 0 | -1 | 0.609 |
| 6 | 1 | 0 | -1 | 0.913 |
| 7 | -1 | 0 | 1 | 0.632 |
| 8 | 1 | 0 | 1 | 0.982 |
| 9 | 0 | -1 | -1 | 0.603 |
| 10 | 0 | 1 | -1 | 0.822 |
| 11 | 0 | -1 | 1 | 0.723 |
| 12 | 0 | 1 | 1 | 0.794 |
| 13 | 0 | 0 | 0 | 0.876 |
| 14 | 0 | 0 | 0 | 0.886 |
| 15 | 0 | 0 | 0 | 0.913 |

The values of regression coefficients were calculated, and the fitted equation (in terms of coded values) for predicting β-glucosidase activity (Y) was as given below regardless of the significance of the coefficients:

Y=0.91+0.16X1+0.064X2+0.023X3+11.269X1X2+0.011X1X3-0.037X2X3-0.042X1^2^-0.091X2^2^-0.069X3^2^

**Tab. 3** Analysis of variance of Box-Behnken design

| Source | SS | DF | MS | F value | Prob>F |  |
| --- | --- | --- | --- | --- | --- | --- |
| Model | 0.32 | 9 | 0.036 | 167.81 | < 0.0001 | significant |
| X1 | 0.21 | 1 | 0.21 | 983.87 | 0.0011 |  |
| X2 | 0.03 | 1 | 0.033 | 154.40 | 0.0857 |  |
| X3 | 4.232E-003 | 1 | 4.23E-003 | 19.86 | 0.0578 |  |
| X1 X2 | 1.102E-004 | 1 | 1.102E-004 | 0.52 | 0.2953 |  |
| X1 X3 | 5.290E-004 | 1 | 5.290E-004 | 2.48 | 0.0195 |  |
| X2 X3 | 5.476E-003 | 1 | 5.476E-003 | 25.70 | < 0.0001 |  |
| X12 | 7.489E-003 | 1 | 7.489E-003 | 35.15 | < 0.0001 |  |
| X22 | 0.035 | 1 | 0.035 | 162.48 | < 0.0001 |  |
| X32 | 0.020 | 1 | 0.020 | 95.25 | 0.0054 |  |
| Lack of Fit | 5.822E-004 | 3 | 1.941E-004 | 0.85 | 0.5330 | Not significant |
| Cor Total | 0.32 | 16 |  |  |  |  |
| R^2^= 0.9954，R^2^_adj_= 0.9895 | | | | | | |

The optimum of location, obtained by differentiation of the quadratic model, for achieving maximal β-glucosidase activity was X1 = 12.0 g/L, X2= 7.05 g/L, and X3 = 10.78 g/L. The predicted optimal β-glucosidase activity corresponding to these values was 1.03 U/mL.

To confirm the goodness of the model for predicting maximal β-glucosidase activity, Additional experiments in triplicates using these optimized fermentation condition were carried out. These triplicate experiments yielded an average maximum β-glucosidase activity of 1.16 U/mL (Tab.4).

**Tab. 4** The β-glucosidase activity after optimization by RSM

| Duplication | β-glucosidase activity (U/mL) | RSD (%) |
| --- | --- | --- |
| 1 | 1.21 | 5.2 |
| 2 | 1.09 |  |
| 3 | 1.18 |  |
| Average | 1.16 |  |

Optimal Medium Composition: maltose, 10.8 g/L; yeast extracts, 12 g/L; peptone, 7.1 g/L; MgSO_4_·7H_2_O, 1.0 g/L; NaCl, 5 g/L; initial pH, 7.0.
